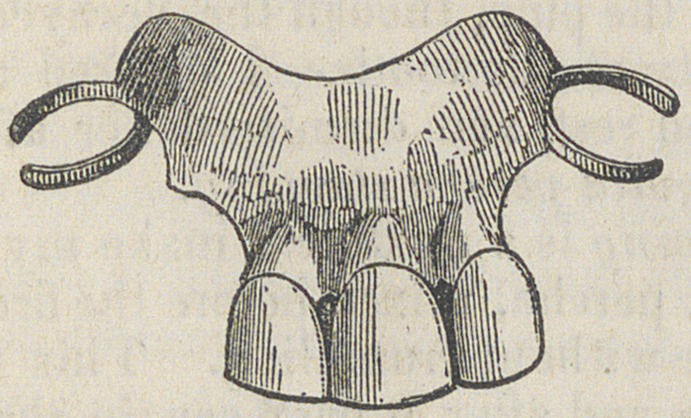# Non-Fatal Accidents from Anæsthetic Agents, with Observations

**Published:** 1854-01

**Authors:** W. H. Mussey

**Affiliations:** Cincinnati


*Non-fatal Accidents from Anæsthetic Agents, with Observations. Read before the Medico-Chirurgical Society of Cincinnati.* By W. H. MUSSEY, M. D.

Recently, in one of the courts of justice in Paris, two surgeons were condemed to pay a fine (merely nominal) for allowing a patient to die under the effects of chloroform. The court sustained the following allegations, and hence its decision.

1st. That chloroform was unnecessarily administered, as the operation to be performed was not of sufficient magnitude to justify its employment.

2d. That the room was not sufficiently ventilated.

3d. That no provision had been made against accident.

In view of the last point, the very natural question arises, What precautions against accidents should be taken by those administering anæsthetic agents?

One answers, that no death has occurred from the use of sulphuric ether, and therefore there need be no apprehension from its administration; another has never *heard* of a death from the chloric ether, and claims for *it* great advantages over other agents; whilst the advocates of chloroform attribute all accidents to the *impurity* of the article.

It is not my purpose to discuss the comparative merits of these agents, but I am persuaded that each, however pure it may be, will meet with idiosyncracies forbidding its administration.

In death from anæsthesia, there is suspension of respiration from paralysis of the nerves presiding over this function, and consequently, suspension of the heart's action. To reanimate, Mr. Jobert de Lambelle (of l'Hotel Dieu) counsels the use of irritants to the skin, currents of air passed over the body, excitants applied to the tongue, cauterization of the mouth and throat with ammonia and currents of electricity. M. Ricord remarked to me that artificial respiration alone, if persevered in, would prevent fatal results, and instanced three cases in his own practice which were saved by that procedure. The following case will illustrate the value of the suggestion.

But first a word as to the agent employed, and means of its administration. We have used for four years the mixture of one part (by measure) of chloroform, and two parts of washed sulphuric ether, both of which are from the manufactory of Messrs. Powers & Weightman, Philadelphia. The vehicle for administration is a large silk handkerchief (an old bandanna) of very loose texture, which has been used for this purpose solely for four years. This is shaken out of its folds and gathered lightly in the hand, and usually a fluid drachm of this mixture is put upon it, and it is held lightly over the face, covering the nose and mouth. If there is much irritation of the lungs, the handkerchief is removed from time to time till it ceases, and when the patient is sufficiently quiet for the commencement of an operation, we not unfrequently add a little fresh material, and leave the handkerchief upon the face for a few minutes.

June 6th, 1852.—Michael O'Hara, native of Ireland, emigrated seven years since; is 27 years old, of sanguine temperament, full habit, capacious chest, great muscular development, and weighs 170 pounds. Once in two or three weeks drinks freely of whiskey for two days, and works steadily in the intermediate time. Had a "spree" for two days last week. Eight months since was working under a bank of earth, which caved upon him and injured his back, since which has had incessant pain in the lumbar portion of the spine, notwithstanding internal medication and the application of cups, blisters, and irritating ointments; now, pressure over the third lumbar vertebra produces pain.

15th.—Propose to apply the actual cautery over the spine.

16th, 8 o'clock, A. M.—At the patient's lodgings, an Irish lad of sixteen years present with us. Patient has eaten nothing for thirteen hours; is lying on his back in a room ten feet square; the head of the bed is under an open window, and at the foot the door stands open. Circulation full and strong, with eighty pulsations per miuute. Commenced the administration of the mixture of chloroform and ether. The first approach of the handkerchief to the face caused slight coughing, which soon subsided. In four minutes, the patient became very loquacious, jabbering in Gaelic, and made great muscular exertion (usually the case with Irish patients), and muscles became rigid; pulse 70. The handkerchief was removed for 30 seconds; the muscles relaxed, the respiration became disembarassed, and pulse 65. Inhalation was resumed and continued for one minute, when I considered the effect nearly sufficient; pulse 60, full and regular.

Putting half a fluid drachm of the mixture upon the handkerchief, I left it upon the face, and stepped below stairs for the heated iron, leaving the lad with the patient. (The iron was in a stove twenty-five yards from the bedside, and I found subsequently, on going over the ground with the same expedition, that I was absent from the room forty seconds.) On my return the wrist was pulseless, and the action of the lungs and heart entirely suspended. I shook the patient, dashed water in his face, turned him upon hir side, then upon the back again, gave various positions to the head, pressed upon the chest to expel the air from the lungs and allow fresh air to replace it. This was kept up for one minute. I then placed myself on the bed at one side of the body, and with my own mouth upon that of the subject, inflated the lungs, then expelled the air by pressure upon the chest, and allowed the pure air to take its place; this in turn was expelled, and the lungs again inflated by my own. In this manner I kept up artificial respiration for the space of three minutes, every alternate inflation being from the atmosphere. Suspending these efforts for a few seconds and seeing no signs of life, I despatched the lad for a professional friend, and resumed artificial respiration. After one minute, I thrust my finger into the throat, and agitated the epiglottis, in hopes to provoke a spasm of the glottis, but without ruccess. Continued artificial respiration for another minute, and a second time thrust my finger into the throat with no better success. Artificial respiration for half a minute, and a third time thrusting my finger into throat, but deeper than before, so as to penetrate between the cords of the glottis, I suddenly withdrew it, but immediately repeating the movement with greater violence, the much wished-for "spasm" grasped my finger, and there was a slight quivering motion of the chest. Artificial respiration resumed, and in two minutes the patient had no need of my assistance.

He was still insensible, but the application of the iron, which was re-heated, partially aroused him. Five minutes after, he was perfectly sensible, complained of great weakness, but had no idea of the peril he had passed through. Half an hour later he seemed as well as patients ordinarily are after the use of chloroform, and subsequently there were no unusual symptoms.

The amount of the mixture used on this occasion was six fluid drachms—two of chloroform and four of ether.

There was no pulsation of the heart, or respiratory movement, for seven minutes.

Reanimation is attributable to artificial respiration and irritation of the glottis.

*Cincinnati, Sept.* 20, 1853.

Since preparing the foregoing, I have witnessed another case of the unfavorable effects of an anæsthetic agent.

Mr. L., farmer, aged 49 years, height five feet ten inches, moderately fleshy, and of lymphatic temperament.

October 12th, 11 A. M.—Patient in the horizontal position; has not eaten for 17 hours; pulse 95 per minute. Commenced the inhalation of the mixture of chloroform and ether. There was slight irritation of the lungs at first, which occasioned some delay. At 11 o'clock, 10 minutes, partial anæsthesia; respiration easy; pulse 80. One minute later, the pulse suddenly dropped to 60, and I removed the handkerchief. There was but little respiratory action; the muscles of the chest were quiet, but those of the abdomen moved feebly. Pulse rose to 18 for a quarter of a minute, and immediately fell to 13. The movement of the abdominal muscles ceased, but only one respiratory movement was lost, as with one hand I opened the mouth, and with the other made pressure upon the chest. Three movements of this kind gave sufficient impulse to the respiration, and no farther assistance was necessary. The pulse steadily improved. A little water was thrown in the face, and the patient returned to consciousness ten minutes after, and for several days he complained of great weakness. Eight days subsequently, with great composure, (without anæsthesia), he submitted to the extirpation of a large tumor of the neck, previous to which I ligated the primitive carotid artery.

Six drachms of the mixture were used.

The facts in this case strengthen the position assumed in connection with the other, viz: that the derangement of the circulation is consequent upon impaired respiratory action.

My Father, Dr. R. D. Mussey, informs me, that a year since, on account of untoward symptoms similar to the above, he was obliged to postpone an operation which was subsequently performed without anæsthesia.

In March, 1840, we were near losing a patient, from the unskillful management of chloroform by a non-professional bystander; and not long after, we tried the mixture of chloroform and ether, and have used no other anæsthetic agent since, under the opinion that the ether sustains the vital powers against the purely sedative effect of the chloroform. Experience has taught us, however, that there is liability to accident from its use.

I am constrained to believe, that a frank avowal of the profession would create astonishment at the great number of non-fatal accidents that have occurred, and that the details would aid in the establishment of principles for conduct in its administration, and serve to throw around the sale and use of anæsthetic agents, such guards as would protect the community from their direful effects. Even in Edinburg, where it is claimed, that in a hundred thousand cases where chloroform has been used, not a single accident has occurred; *untoward symptoms have compelled the suspension of the use of the article*, (as I was informed by an assistant of Dr. Simpson), which the operators profess to be able to trace to the *impurity* of the agent employed.

My attention has just been called to the researches of Prof. Horsford, of Cambridge, Mass., (*Bost. Med. and Sur. Journal*, Oct. 19), and to a paper in the *Am. Journal of Medical Science* for Oct., from Dr. Bickersteth, of Liverpool, to which I refer as corroborative of the positions herein assumed, viz:

*First.* That accidents from anæsthesia are generally attributable to idiosyncracies in the subjects.

*Second.* That derangements of the circulation depend upon the disturbance of the function of respiration.

*Third.* That artificial respiration is the most valuable of all means for counteracting dangerous symptoms.—*W. Lancet.*

Oct. 25, 1853.

*A Plate of Artificial Teeth swallowed, and subsequently discharged per Anum.* By W. H. Mussey, M. D.—Mr. E. S., of Fulton, was awakened at midnight, of June 14th, 1853, by a distressing sensation in the throat, and discovered that a plate of three teeth, which he wore in the upper jaw, had disappeared. Successive acts of deglutition did not relieve the oppression, and Dr. E. H. Ferris was called, who employed such means as were at his command to clear the œsophagus, but emetics, &c., affording no relief, they called on me at 2 A. M., the 15th.

The patient insisted that the missing teeth were in the œsophagus, at a point corresponding to the last cervical vertebræ. In using the various œsophageal instruments, there was no difficulty in passing them below the point indicated, and that without contact with anv foreign body. Instruments were employed for twenty minutes, at intervals of three or four minutes, when the question of the expediency of œsophagotomy was considered. In order to assure myself of the presence of a foreign body, I again introduced a slender instrument, having a slightly-hooked extremity. The exploratory movements caused some irritation, and a vigorous spasm of the œsophagus. The withdrawal of the instrument was followed by a more violent spasm, and the sense of obstruction was relieved: the patient exclaimed, he had "swallowed the teeth!"

The use of bulky food, as mush, rice bread, and potatoes, was advised, and a patient waiting for developments.

June 21st.—At 8½ o'clock, A. M., (six and one-third days after the accident), without any previous unpleasant symptoms, there passed the anus, attended with slight pain and a few drops of blood, the set of teeth, of which the accompanying cut is a representation.

To this day, the patient has not had the slightest derangement of his system.

Cincinnati, Oct., 1853.

## Figures and Tables

**Figure f1:**